# Dissociating Sensory and Motor Components of Inhibition of Return

**DOI:** 10.1100/tsw.2006.172

**Published:** 2006-07-27

**Authors:** Anne B. Sereno, Cameron B. Jeter, Vani Pariyadath, Kevin A. Briand

**Affiliations:** Department of Neurobiology and Anatomy and the Keck Center for the Neurobiology of Learning and Memory, University of Texas — Houston Medical School, Houston, TX 77030, USA

**Keywords:** spatial attention, IOR, saccade, pointing

## Abstract

Two explanations for inhibition of return (IOR) have been proposed. The first is that IOR reflects inhibition of attentional processing at previously cued locations, resulting in altered sensory analysis. The second is that IOR reflects the inhibition of responses directed towards those previously cued locations. We used a variant of a double-saccade paradigm to dissociate these two proposed effects of IOR and attempted to reveal both effects within the context of a single experimental task. Subjects viewed a series of exogenous cues and then made a localization response to subsequent targets with either a target-directed saccade or a pointing response. Results were similar for both response modes. An important finding was that the pattern of IOR depended critically on how subjects reacted to the exogenous cues. Subjects either oriented to the cued locations (via saccades or pointing) prior to responding to the target (Respond), or passively viewed the cues before responding (Ignore). In the Respond condition, IOR was observed at the most recently cued position. Although this could be consistent with an altered sensory interpretation, it would also be consistent with a spatiotopic representation. In the Ignore condition, the sole inhibited location was not the most recently cued position, but the first cued position. This finding is surprising and in conflict with previous work with multiple exogenous cues. The data are discussed in relation to a number of prominent issues in the area of IOR and suggest important new constraints and boundary conditions.

## INTRODUCTION

When an exogenous cue is flashed in the visual field, there are two well-documented phenomena that follow. Within approximately 50–150 msec of cue appearance, responses to visual targets that appear in the cued location are facilitated. This effect has been documented for a variety of different responses including detection, localization, and discrimination of nonspatial features[1,2,3,4,5,6], and has been observed for both manual and saccadic responses[2,3,6]. The facilitation effect is followed at longer cue-target intervals (stimulus onset asynchronies, or SOAs) of 200 msec or more by an opposite inhibitory effect known as inhibition of return, or IOR. IOR has also been reported for a variety of different classification responses and response modes[5,7,8,9,10,11,12,13,14,15].

IOR has been found in controlled experiments with simple visual displays, as well as in situations with higher degrees of ecological validity[16]. It is widely believed that IOR functions to facilitate visual search. Without some way of keeping track of where we have previously, but unsuccessfully, inspected, visual search would be an inefficient process. IOR has been proposed as a means of enhancing visual search by preventing the reinspection of recently attended locations[1,17,18,19,20], thereby creating a bias towards searching new spatial regions.

This functional explanation for IOR leaves unclear the nature of the underlying processes. The specific question we address in this paper concerns what happens after IOR has been generated. In other words, what exactly is being inhibited in IOR? Explanations of which processes are implicated in IOR tend to fall into two general categories[21]. According to the first account, what we observe as IOR is actually a suppressed orienting of covert attention to previously cued or attended locations. A delayed or weakened reorienting of attention to these locations would result in poorer processing of any stimuli that subsequently occurred there. There would thus be a less-efficient analysis of sensory events at previously cued or inspected locations. We will henceforth refer to this alternative as the sensory/attentional account of IOR. Taylor and Klein[15] and Klein[22] reviewed evidence in favor of this approach. For example, (1) the same factors (e.g., target modality and intensity) influence both the initial facilitation and later IOR in response to spatial cues[9], and (2) IOR can be observed for nonspatial target features[5,10,11,12]. This latter observation is consistent with a delayed or weakened attentional response, since this would affect the analysis of all sensory features for a target stimulus, a finding which has been further confirmed by signal detection analysis in an IOR paradigm[23].

In contrast to the sensory/attentional approach, a second view has stressed motor or overt response factors in IOR. It has been argued that the act of allocating covert attention in space has close functional links to the act of physically orienting towards spatial locations[24,25]. If the sensorimotor systems involved in orienting attention to stimuli are the same ones involved in overtly responding to spatial stimuli, then exogenous cues could thus either facilitate (at short SOAs) or inhibit (at longer SOAs) the *responses* to targets directly, rather than affect early sensory analysis. Indeed, it has been argued[26] that IOR reflects a bias against overtly responding to inhibited locations. For example, such an IOR mechanism might take the form of an inhibited motor plan for an eye movement to previously cued positions. Evidence consistent with this approach also exists[7,15,21,22,27].

Evidence in the literature supports each approach. In fact, some recent studies have suggested that both SA and motor processes are required in order to explain IOR[13,15]. Each of these studies reported evidence that was consistent with both alternatives. However, while both sensory and motor IOR effects were observed in these studies, these effects were seen in separate blocks of trials, tasks, or testing sessions. In the present paper, we used a paradigm where the possible sensory and motor factors influencing IOR might be revealed within a single task or session, eliminating possible confounds due to task differences.

Specifically, we used a modified double-saccade task, first described by Hallett and Lightstone[28]. The double-saccade task has been used in physiological studies in primates to dissociate sensory and motor responses of neurons. In a simple target-elicited saccade paradigm, a monkey is trained to make an eye movement to a visual target as activity in neurons is observed. However, a cell may be firing only because a stimulus appears within its receptive field (sensory related). On the other hand, some of this activity may be due to preparation to make a saccade to the stimulus location (motor related). What needs to be determined is whether the cell's activity is caused by sensory or motor factors. The double-saccade task represents an elegant attempt to dissociate these two very different effects. In this task, two cues are flashed in quick succession, then subjects make saccades to remembered cue positions. The rationale for this procedure is that after the first eye movement is completed, there is a dissociation between the retinotopic location stimulated by the second cue (sensory), and the retinotopic location corresponding to the vector or destination of the second planned eye movement (motor). The ability of the double-saccade task to differentiate between sensory and motor effects critically depends on the fact that the superior colliculus, as well as other cortical areas considered important for eye movements and spatial representation (e.g., LIP, FEF), have been found to use a predominately retinotopic representation, i.e., activity depends on the retinal location of the cue position or movement goal with respect to *current* fixation[29,30,31,32]. Hence, although Posner and Cohen[1] showed that IOR could be mapped in spatiotopic coordinates, no physiological evidence for a stable spatiotopic representation was found in those areas considered important in eye movements, attention, and spatial representation. If IOR occurs in a retinotopic sensory or motor representation, a double-saccade task should be able to dissociate sensory and motor effects. As illustrated in Fig. 1, if IOR is caused by sensory cues presented in locations c1 and c2 in the left visual field (see Fig. 1A), and this sensory activation modulates the response of sensory neurons in superficial layers of the superior colliculus (S1 and S2, Fig. 1C), then IOR should be present for subsequent targets presented in locations 1 and 2 (Fig. 1B). If IOR is caused by the motor programming and execution of saccades (m1 and m2, Figs. 1D and 1E) and this motor activation modulates the response of saccade-related neurons in intermediate and deep layers of the superior colliculus (M1 and M2, Fig. 1F), then IOR should be present for subsequent targets presented in (i.e., movements to) locations 1 and 3 (Fig. 1B).

Mays and Sparks used this procedure to dissociate sensory and motor responses of cells within the superior colliculus (SC)[29]. Specifically, Mays and Sparks reported that for most saccade-related neurons, direct retinal (i.e., sensory) activation of the overlying superficial visual neurons had no effect on either the vigor or probability of a saccade-related (i.e., motor) discharge. They concluded that the discharge of overlying visual cells is neither necessary nor sufficient to activate most saccade-related cells. Put another way, motor-related discharge in most saccade-related SC cells is observed independently of any sensory activation.

Mays and Sparks' ability to dissociate sensory and motor factors in collicular neurons takes on added significance in the present context, since the SC has been implicated as crucial for generation and/or maintenance of IOR. Early behavioral work suggested that midbrain structures played a role in IOR[7,8,33]. More direct evidence recently has confirmed this proposed relationship by demonstrating that patients with lesions of the SC show deficiencies in IOR[34,35,36]. Finally, neurophysiological work has also shown that the SC is involved in some critical aspects of IOR[37,38]. Thus, assuming that the SC plays a role in IOR and that we have some knowledge about sensory and motor responses in the SC in a double-saccade paradigm, we used the double-saccade paradigm (cues and responses to those cues) to generate IOR. Then, after a delay of 1000 msec, we presented a target signal that was intended to reveal the consequences of IOR (when present). We then assessed the effect of IOR in positions that either should have generated sensory responses, generated motor responses, or not have been activated (neutral condition). In this way, we were able to separate sensory effects from motor effects of IOR. This assumes that if the generation of activity occurs in specific neurons (either sensory or motor), it is these same cells that will show an effect (if present). This is the simplest assumption — that a sensory-generated effect will affect subsequent sensory processing and a motor effect will affect subsequent motor processing.

To summarize, (1) there is a continuing debate over the role of sensory and motor factors in IOR; (2) a specific paradigm, the double-saccade task has been previously used to dissociate visual and saccade-related neural responses in the SC; and (3) the SC appears to be critical for the generation of IOR. For these reasons, we carried out a series of experiments using a variation of a double-saccade task to dissociate and measure the independent contributions of sensory and motor influences of IOR. We examined these separate sensory and motor contributions to IOR within the context of a single task and testing session. This design controls for possible confounding or uncontrolled strategic factors that may influence performance when sensory and motor effects are revealed only in separate blocks of trials, sessions, or tasks.

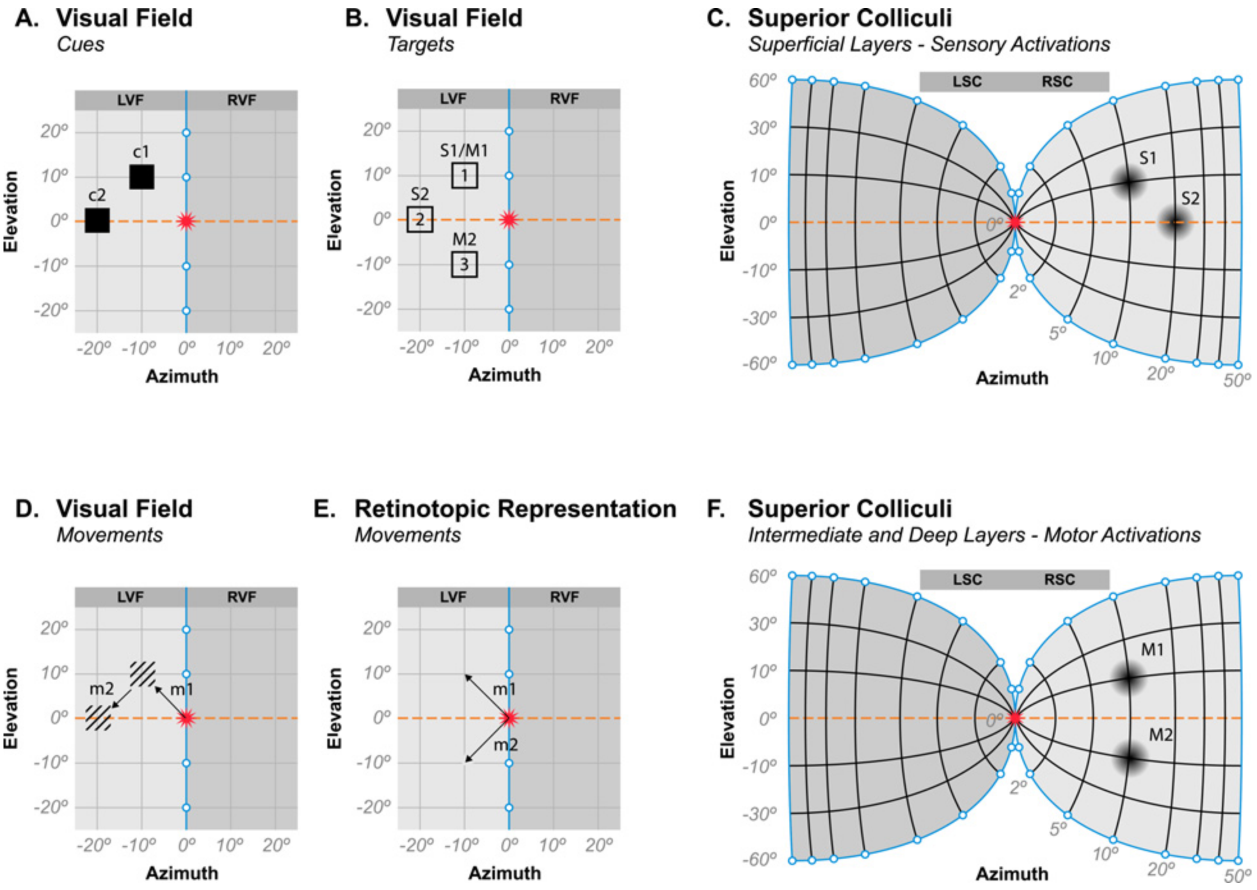

**FIGURE 1.** Double-saccade paradigm and the SC. (A) Visual field (spatiotopic) representation of cues in the double-step paradigm. Vertical meridian is indicated by the blue line with open circles (—o—o—). Horizontal meridian is indicated by the orange dashed line. The right visual field (RVF) is identified at the top of each panel and distinguished from the left visual field (LVF) by a slightly darker background gray. Two locations in the left visual field are labeled c1 and c2 and indicate the positions of the first and second cue, respectively, when the eyes are fixating the central fixation point (red star at center of display). (B) Visual field representation of possible subsequent target or probe positions. In an IOR paradigm, the initial presentations of cues are followed by a target. This panel indicates 3 possible locations of the target (1, 2, and 3) that are critical to dissociate sensory and motor effects. Notice that 1 and 2 are in the same positions as the cues, c1 and c2, respectively, in panel A. The third possible target location, 3, is located to the left and below fixation (–10° azimuth and –10° elevation). The importance of these positions will be discussed at the end of this caption. (C) Schematic representation of sensory activations, S1 and S2, in superficial layers of the SC to cues (depicted in panel A) presented in locations c1 and c2, respectively. Vertical and horizontal meridians are indicated and depicted as described in panel A. The left SC (LSC) and right SC (RSC) are identified at the top of the figure. Note the expansion of the foveal region representation in the colliculus as well as the reversal of the representation (i.e., the RVF in darker gray is represented in the LSC, also in darker gray). (D) Visual field (spatiotopic) representation of saccades to remembered cues. Because the two cues are no longer present, their locations are indicated by diagonally filled squares. The arrow labeled m1 represents the first saccade from fixation to the remembered location of the first cue, c1. The arrow labeled m2 represents the second saccade from the remembered location of the first cue, c1, to the remembered location of the second cue, c2. (E) Retinotopic representation of saccades to remembered cues. The arrow labeled m1 represents the first saccade from fixation to the location of the first cue, c1. The arrow labeled m2 represents the second saccade from the location of the first cue, c1, to the location of the second cue, c2, remapped in retinotopic coordinates whereby the location of the first cue, c1, is now centered at fixation (red star at center of display). (F) Schematic representation of saccade-related neuron activations, M1 and M2, in the intermediate and deep layers of the SC coupled to the onset of a saccade to the remembered location of the first cue, c1, and to the saccade to the remembered location of the second cue, c2, respectively. Vertical and horizontal meridians are indicated and depicted as described in panel A. Note that the location of saccade-related activations for the eye movement to the second cue, M2 (see panel F) lies in a different part of the SC map than the location of sensory related activations for the second cue, S2 (see panel C). Hence, after the presentation of cues and execution of the double saccade, if a subsequent target is presented at target location 1 in panel B (i.e., same visual field location as c1 in panel A and same motor field location as m1 in panel E) and behavioral inhibition is found, this inhibition can be the result of either sensory (S1, see panel C) or motor (M1, see panel F) activations. However, if a subsequent target is presented at target location 2 in panel B (i.e., same visual field location as c2 in panel A) and behavioral inhibition is found, this inhibition can only be the result of sensory (S2, see panel C) activations. Note that no cue-related activation for the second cue, c2, appears for saccade-related neurons in the intermediate and deep layers (see panel F). Most saccade-related neurons in the intermediate and deep-layers as defined by Mays and Sparks[29] have no, or very weak, visual responses. However, most saccade-related neurons do discharge for a saccade in the absence of a visual target (or visual stimulation of overlying visual cells). Hence, there is activation, M2 (panel F), for the second saccade, m2 (panel E), made to the location of the remembered second cue (note that this activation is in remapped retinotopic coordinates). Thus, after the presentation of cues and execution of the double saccade, if a subsequent target is presented at target location 3 in panel B and behavioral inhibition is found, this inhibition can only be the result of motor (M2, see panel F) activations.

## EXPERIMENT 1

We will first describe the general procedure followed in these experiments. As shown in Fig. 2, subjects viewed a display composed of a central fixation spot and 8 peripheral spots positioned equidistantly in a circular pattern. The display was oriented such that one of the spots always occupied the vertical (12 o'clock) position. Two of these peripheral locations were briefly brightened in sequence, with the two cue positions always 90º apart in the circular display. Following the two peripheral cues, the fixation spot brightened to return attention to fixation. All 16 possible cue sequences, including both clockwise and counterclockwise cue directions, were shown to subjects. Following a brief interstimulus interval (ISI), one of the 8 peripheral locations changed from gray to green and subjects had to then make a saccadic localization response to the green target as quickly as possible.

In addition to trying to dissociate sensory and motor influences on IOR, we also investigated the influence of one potentially important variable. In a typical IOR paradigm, subjects view one or more exogenous cues passively and then make some response to a subsequent visual target. That is, subjects are not required to explicitly respond to the cues. However, two previous studies have directly compared this type of cueing procedure to one where subjects made saccades to both cues and targets[15,21]. Interestingly, data from both studies showed that IOR was greater when responses had to be generated to both cue and target, as compared to the standard cueing procedure. Thus we felt it important to manipulate the nature of the subject's responses to the cues in the present paradigm. While our experimental procedures may be able to dissociate sensory and motor effects of IOR, based on these previous data, it seemed likely that the magnitude of IOR observed would be enhanced if subjects responded to the exogenous cues. Accordingly, all of our subjects were tested under two different conditions. In the Respond condition, after the cue sequence was completed, subjects had to make saccades to each of the cued positions in the correct sequence before viewing and responding to the target. In a second condition (Ignore), they *passively* viewed the exogenous cues before responding to the target.

### Methods

### *Participants*

Eighteen Rutgers University students were tested in a single testing session consisting of two different experimental conditions. Subjects received either partial course credit or monetary compensation for participating. All had normal or corrected vision. All experiments reported herein were approved by the institutional review board for human subjects at Rutgers University, the University of Texas at Houston Medical School, and Rice University. Informed consent was obtained from all subjects prior to testing and subjects were debriefed following completion of the experiment.

### *Stimuli and Apparatus*

All stimuli were shown on a 17-inch, multisynch monitor having a refresh rate of 75 Hz. The stimulus display consisted of a circular array of 8 gray spots plotted on a black background. These were positioned equidistantly from one another at an eccentricity of 7.5º from a central fixation spot. This central spot, as well as the 8 peripheral ones, was 1º in diameter. The display was oriented such that one of the spots was located in the vertical (12 o'clock) position. When a cue was shown, it consisted of a brief, 40-msec

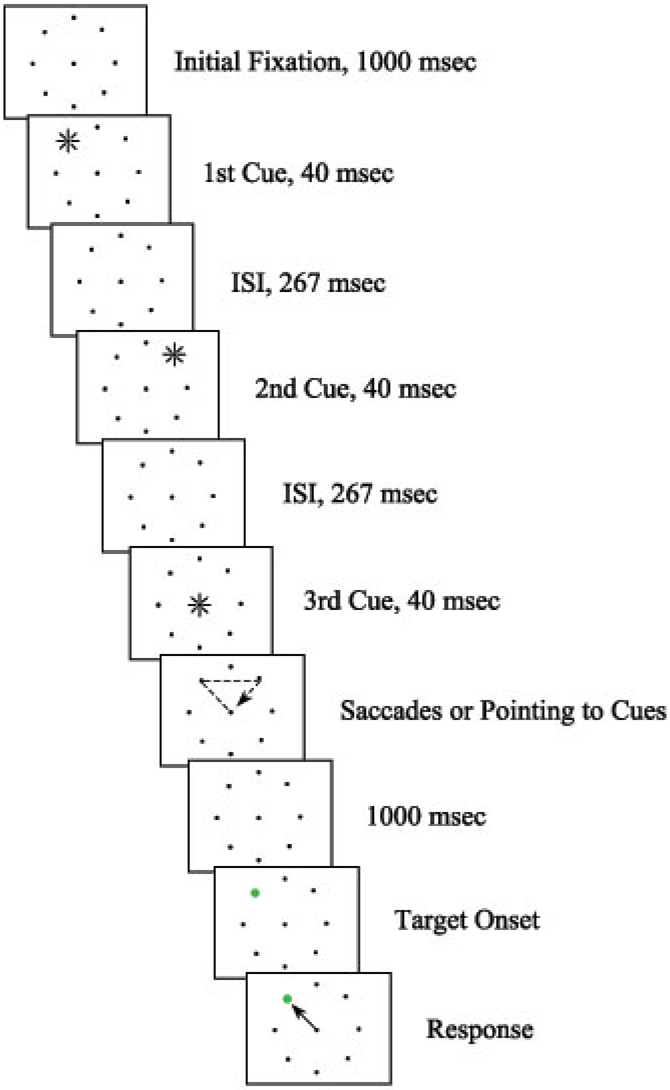

**FIGURE 2.** Sequence of events during each trial in the Respond condition. For the Ignore condition, the sequence was identical with the exception that no responses to the cues were required.

change in the color of one of the spots from gray to white, and then back to gray. For the target stimulus, one of the 8 peripheral spots was switched from gray to green, and remained in view either until the subject had responded or until 1000 msec had elapsed.

Subjects had to make a speeded eye movement to the target. Eye movements were recorded using an ISCAN RK-426 eye-tracking system, interfaced with an infrared sensitive camera. For saccades to the targets, a saccade was assumed to have started when eye velocity exceeded 120º/sec, and the end of the saccade was indicated when velocity fell below 8º/sec. The terminal point of the saccade had to be within 2.5º of the cue or target position to be scored as correct. Saccades that terminated in any other position on the screen were coded as errors.

## Procedure

Each trial followed a sequence similar to that illustrated in Fig. 2. After the subject had oriented to the central fixation point by fixating the center dot, the trial sequence started. Following a timed fixation period (1000 msec), a cue sequence was initiated, which consisted of a 40-msec brightening of one of the 8 peripheral locations, followed, after an ISI of 267 msec, by the brightening of a second peripheral location. The position of the second cue was always 90º away from the position of the first cue, either clockwise or counterclockwise in the circular array. The second cue was shown for 40 msec, and, after another 267-msec ISI, the central fixation spot then brightened for 40 msec. Subjects had to remain fixated during the entire cue sequence. If a saccade was initiated, the trial was terminated and replaced in the pool of unfinished trials.

Following the cue sequence, two procedures were followed. In the Respond condition, following the cue sequence, subjects had to make a series of three saccades to the locations of the first, second, and third cues in the correct order. The criteria to determine initiation, conclusion, and accuracy of these cue-directed saccades were identical to those used for target-directed saccades (eye velocity above 120º/sec, etc.). Failure to generate the correct eye movement sequence within 2.5 sec resulted in the trial being cancelled and being replaced in the pool of unfinished trials. Following successful replication of the cue sequence, a 1000-msec ISI was presented followed by a target in one of the 8 locations. Subjects had to make a speeded saccade to this target. In the Ignore condition, the cue sequence was followed by a 1000-msec ISI and then by a target stimulus at one of the 8 peripheral locations. Subjects only made a speeded saccade to the target, and did not respond to the cues.

The Respond and Ignore conditions were presented in separate blocks of trials, with their order counterbalanced between subjects. Within each condition, there were 16 possible cue sequences (8 possible starting positions and 2 possible directions, clockwise or counterclockwise). For each of these 16 sequences, there were 8 possible target locations. Thus there were 128 possible cue-target sequences, each presented once per condition. The entire testing session took approximately 45 min.

## Statistical Analysis

To separate the possible contributions of sensory-based and motor effects of IOR, data were analyzed in the following manner (see Fig. 3A). The position of the first cue was designated as location 1 and that of the second cue as location 3 (ignoring the particular cue sequence and whether the cues occurred in a clockwise or counterclockwise pattern). All of the other 6 locations were then numbered consecutively. All cue sequences were normalized and collapsed onto a single schematic representation as illustrated in Fig. 3A.

The sensory/attentional- and motor-based explanations of IOR make different predictions concerning which locations should be inhibited in this paradigm. The sensory/attentional model (Fig. 3B) predicts that IOR should only occur at locations 1 and 3 (designated S1 and S2), since they are the only positions that were retinally stimulated while the subject was fixated on the central position. On the other hand, the motor model (Fig. 3C) predicts that IOR should occur at locations (on vectors with respect to fixation) to which saccades were executed or planned (M1, M2, and M3). For example, location 1 (designated M1) should be inhibited under the motor model, since the first saccade was executed to the first cued position.

Also, locations 4 and 7 (designated M2 and M3) might also be inhibited. Even though no sensory event occurred at these positions, M2 and M3 represent the approximate positions to which motor responses would have been made to the second and third cues, respectively. That is, the predicted IOR effects for responses to targets are plotted retinotopically with respect to current gaze position at fixation (i.e., the position of the eye movement goal at eye movement onset). Thus if previously executed or planned motor responses are inhibited, responses to targets in positions M1, M2, and M3 should be slowed.

The reader will note that of the 8 possible target positions, only 4 of them are predicted to show possible IOR effects by the above analysis. The remaining 4 positions (locations 2, 5, 6, and 8) served as a neutral baseline for purposes of evaluating IOR at each of the 4 critical target locations, via planned contrasts.

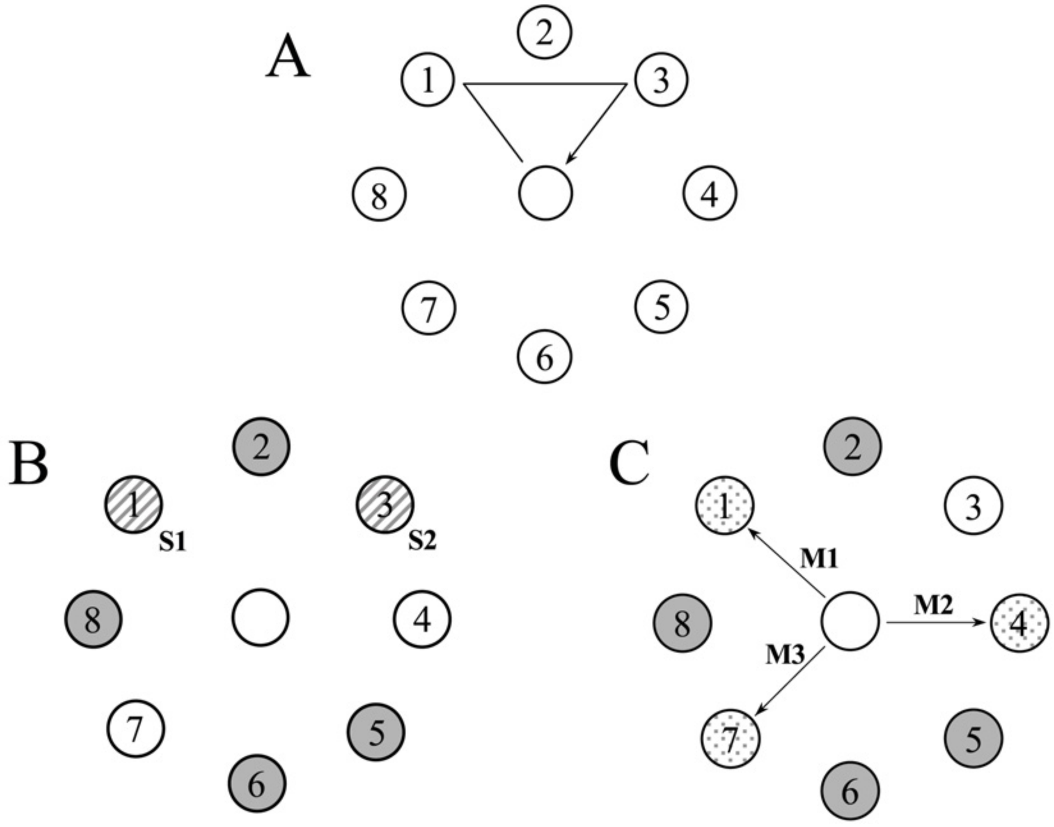

**FIGURE 3.** Examples illustrating the data-analysis procedures. (A) Starting with the position of the first cue, positions are numbered sequentially in the direction of the second cue. The first cue is always in position 1 and the second cue in position 3. (B) With the subject's gaze at fixation, the sensory account of IOR predicts that inhibition should only be observed at positions where cues occurred (S1 and S2, indicated with a diagonal fill pattern). (C) The motor account of IOR predicts that responses along the vectors representing previously executed or cued movements should be inhibited. Thus, IOR should be observed at M1, M2, and M3, indicated with a dotted fill pattern. Note that IOR occurring at position 1 is ambiguous, since both the sensory and motor accounts predict IOR there. Finally, positions 2, 5, 6, and 8 (shaded) are neutral, since neither account predicts IOR at these locations.

## Results

Each subject's median response times (RTs) were calculated for targets appearing in each position (1 through 8). The RTs for the Respond and Ignore conditions were first analyzed by analysis of variance (ANOVA), with Condition (Respond/Ignore) and Target Position (1-8) as factors. Separate ANOVAs for the Respond and Ignore conditions then served as the basis for planned contrasts evaluating the IOR effects, with the Mean Square Error terms from these separate analyses used as the error term for the planned contrasts. IOR effects were determined by planned contrasts wherein RTs to targets in the four critical locations (S1/M1, S2, M2, and M3) were compared to RTs to targets appearing in the remaining four neutral positions. Fig. 4 shows the summarized data included in our schematic-stimulus display to facilitate interpretation. The 4 neutral locations used as a baseline for the planned contrasts are shaded in gray. For consistency, IOR effects in the text will be specified as *positive* values (e.g., $RT_{S1} > RT_{Neutral}$).

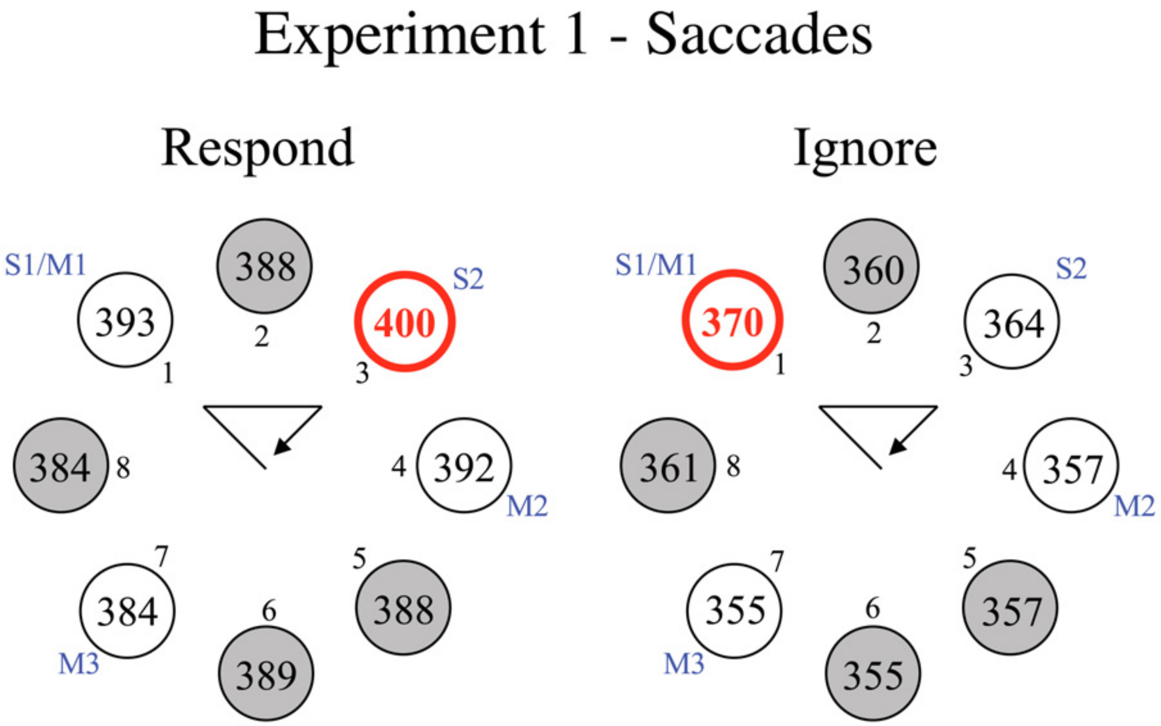

**FIGURE 4.** Mean of median saccadic RT in the Respond and Ignore conditions of Experiment 1. The mean RTs to targets occurring in each of the 8 positions are given. The arrow schematically illustrates the sequence of cues (position 1, then 3, then fixation). The shaded circles are the neutral locations. Significantly inhibited locations are indicated in red.

The overall ANOVA showed a significant effect of Condition, $F_{1, 17} = 11.85$, $p < 0.004$ (RTs of 390 and 360 msec for the Respond and Ignore conditions, respectively). There was a significant effect of Target Position, $F_{7, 119} = 2.85$, $p < 0.01$. The Condition × Target Position interaction was not significant ($F_{7, 119} < 1$).

**Respond Condition – Planned Contrasts:** Planned contrasts evaluating inhibition at S1/M1, S2, M2, and M3 were conducted using the Mean Square Error terms from separate ANOVAs of the Respond and Ignore conditions. There was significant IOR at position S2 (13 msec, $F_{1, 119} = 8.69$, $p < 0.004$; MSE = 282.364; indicated in red in Fig. 4). However, IOR was not significant at position S1/M1 (6 msec, $F_{1, 119} = 1.54$, $p > 0.21$). There was no evidence for IOR at either position M2 or M3 (5 and –3 msec, respectively, both Fs < 1, $p > 0.32$). **Ignore Condition – Planned Contrasts:** For the Ignore condition (MSE = 266.889), position S1/M1 showed significant IOR of 12 msec, $F_{1, 119} = 7.41$, $p < 0.008$ (indicated in red in Fig. 4). Inhibition was nonsignificant at position S2 (6 msec, $F_{1, 119} = 2.05$, $p < 0.16$). Finally, there was no hint of IOR at either position M2 (–1 msec, $F < 1$, $p > 0.81$) nor at M3 (–3 msec, $F_{1, 119} < 1$, $p > 0.49$).

Analysis of error data revealed that neither the main effects nor their interaction were significant (all Fs < 1). Planned contrasts on these also revealed no significant effects (see Table 1).

**TABLE 1**
**Error Rates (in %) for Localization Responses to Targets in Experiments 1–4**

| | Neutral | S1/M1 | S2 | M2 | M3 |
|---|---|---|---|---|---|
| **Experiment 1** | | | | | |
| Respond | 2.0 | 1.0 | 1.4 | 1.0 | 1.4 |
| Ignore | 1.6 | 1.4 | 2.1 | 1.7 | 1.7 |
| **Experiment 2** | | | | | |
| Respond | 0.1 | 0.3 | 0.0 | 0.0 | 0.0 |
| Ignore | 0.1 | 0.0 | 0.3 | 0.0 | 0.0 |
| **Experiment 3** | | | | | |
| Respond | 0.8 | 0.9 | 2.1 | 1.5 | 1.8 |
| Ignore | 1.4 | 1.4 | 1.1 | 1.1 | 2.4 |
| **Experiment 4** | | | | | |
| Ignore Long | 5.3 | 6.3 | 5.2 | 4.2 | 4.5 |
| Ignore Short | 5.2 | 4.5 | 3.5 | 3.1 | 7.3 |

## Discussion, Experiment 1

In Experiment 1, we used a modified double-saccade task in order to dissociate sensory and motoric influences of IOR. There were three main findings from this manipulation. First, we obtained evidence for a sensory factor in IOR in the Respond condition, since IOR was found only at position S2. Given that no response was generated or prepared to position S2, the IOR obtained at this position should reflect sensory or attentional factors rather than motoric ones. In contrast, when we tested subjects in the Ignore condition, the only evidence for IOR was at position S1/M1. This was unexpected and surprising, as many previous studies have demonstrated that IOR is strongest for the most *recently* cued spatial location, not the first or earliest cued location[19,20,39,40,41]. Further, the IOR we obtained at this position is ambiguous and could reflect either sensory or motor factors. Finally, instructing subjects to overtly respond to the exogenous cues did not simply affect the magnitude of IOR as we had expected, but changed which locations showed inhibition. This is an important finding which we will return to in further experiments.

## EXPERIMENT 2

In Experiment 2, we examined whether a *manual* mode of responding resulted in a pattern similar to that observed in the first experiment. We replicated these procedures with a separate group of subjects, replacing the saccadic localization task with a manual response. Although the vast majority of studies investigating IOR with manual localization responses have relied on key press responses, IOR has also been studied using goal-directed or reaching responses[3,6,42]. In addition, recent work has reported that the SC also contains many neurons with activity related to goal-directed arm movements[43,44,45,46]. We felt that the paradigm we are using should also be able to dissociate sensory and motor influences of IOR when manual goal-directed responses were used. Accordingly, in Experiment 2, all responses (both to cues and targets) required subjects to *point* to locations on the stimulus display screen.

## Methods

### Participants

Eighteen Rutgers University students were tested in a single testing session consisting of two different experimental conditions. Subjects received either partial course credit or monetary compensation for participating. All had normal or corrected vision, and informed written consent was obtained from each participant. None of the subjects in Experiment 2 participated in the previous experiment.

### Stimuli, Apparatus, and Procedure

The stimuli and procedure used were essentially the same as that for Experiment 1. A touch screen (Keytek MagicTouch) was overlaid on the display screen and subjects sat approximately 35 cm from the screen. Instead of measuring eye movements, subjects were required to touch the fixation point on the display screen with a stylus, and then move it to the cue or target position. The stylus had to be placed within 2.3° of the center of the cue or target position in order to be counted as a correct response. Following cue or target appearance, a response was assumed to have occurred when the stylus was lifted from the touch screen and this latency was recorded. Since the pointing response required subjects to be within comfortable reaching distance of the display screen, stimuli for the pointing version of the manual response were scaled down so that they would cover the same retinal area as they did when viewed at a greater distance (45% of normal size). In all other respects (stimulus sequence, number of trials, etc.) the procedure used was identical to that used for Experiment 1.

When subjects pointed to the touch screen with their fingers, we found that their hand could obscure the lower part of the stimulus display. To circumvent this problem, we used a stylus (approximately 8 in. long). Subjects held the stylus near the middle, which allowed them to hold their hand further from the screen (i.e., it was held more like one would hold a paint brush than the way one would hold a pencil). This procedure allowed subjects to perform the task with minimal disruption of the stimulus display while still allowing for accurate responses. Indeed, as indicated below, the similarity of the results obtained using saccadic (Experiments 1 and 3) and manual responses suggests that this was not a significant factor in our experiments.

## Results

Data were analyzed as in Experiment 1. Each subject's median RT was calculated for targets appearing in each of the 8 possible positions, for both the Respond and Ignore conditions. Fig. 5 shows the summarized mean of median reaction times for all subjects. A preliminary ANOVA with Condition (Respond/Ignore) and Target Position (1-8) as factors showed a significant effect of Condition, $F_{1, 17} = 4.51$, $p < 0.05$ (RTs of 407 and 392 msec for the Respond and Ignore conditions, respectively). Neither Target Position ($F < 1$, $p > 0.65$) nor the Condition × Target Position interaction ($F_{7, 119} = 1.39$, $p > 0.22$) were significant.

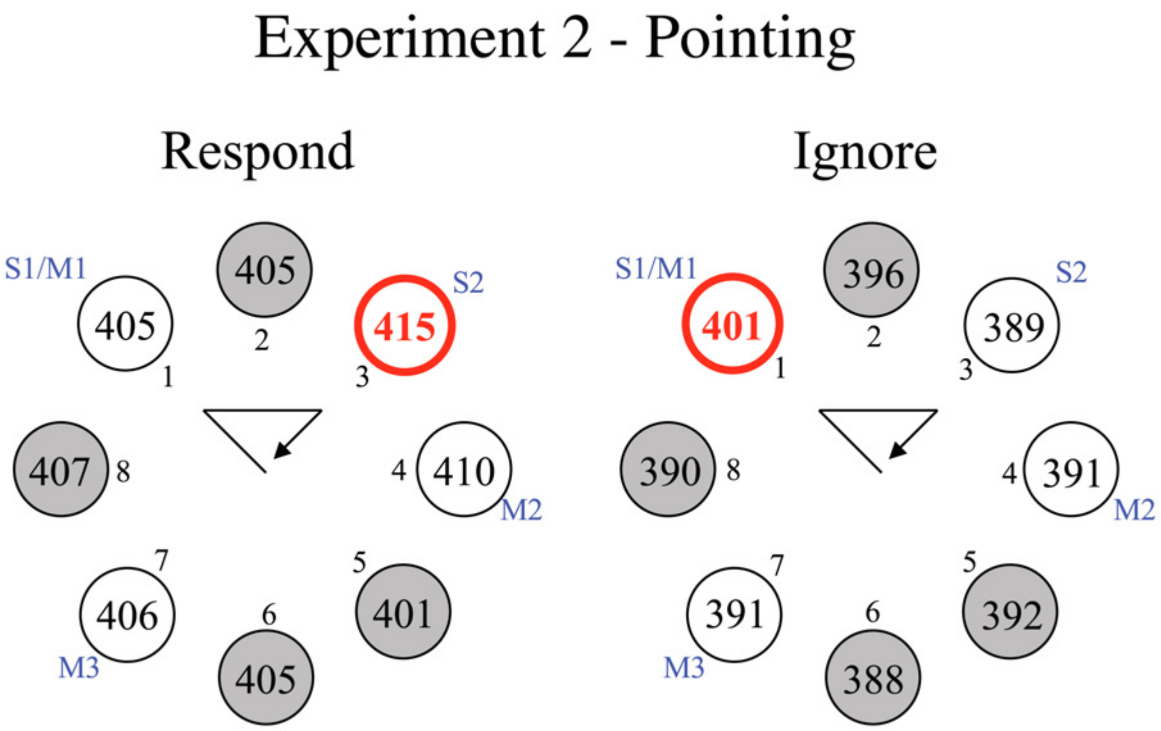

**FIGURE 5.** Mean of median manual RT in the Respond and Ignore conditions of Experiment 2. Formatting is the same as in Fig. 4.

**Respond Condition – Planned Contrasts:** Planned contrasts were conducted using the Mean Square Error terms from separate ANOVAs of the Respond (MSE = 375.298) and Ignore conditions (MSE = 242.733). In the Respond condition, as was seen in Experiment 1, there was significant inhibition at position S2 (11 msec, $F_{1, 119} = 4.39$, $p < 0.04$; indicated in red in Fig. 5). Again, similar to Experiment 1, there was no inhibition at position S1/M1 (1 msec, $F_{1, 119} < 1$, $p > 0.84$). Finally, there was no inhibition at either of the two motoric locations M2 (6 msec, $F_{1, 119} = 1.25$, $p > 0.26$) or M3 (2 msec, $F < 1$, $p > 0.81$). **Ignore Condition – Planned Contrasts:** For the Ignore condition, responses to targets appearing at position S1/M1 were again significantly inhibited (9 msec, $F_{1, 119} = 5.13$, $p < 0.03$; indicated in red in Fig. 5). There was no IOR at either position S2, M2, or M3 (–3, –1, and –1 msec, respectively, all Fs < 1, $p > 0.52$ for all). Analysis of error data revealed no significant effects (see Table 1).

## Discussion, Experiment 2

Results from the first two experiments were somewhat surprising and not entirely consistent with the rationale we originally laid out. We expected that the critical factor affecting where we obtained IOR would be that of target location; i.e., would the inhibited locations be those that suggested poor sensory analysis, or those consistent with inhibited responses? Instead, we observed that the most critical factor influencing which locations were inhibited was whether the subject needed to respond to the exogenous cues. Using both saccadic (Experiment 1) and manual responses (Experiment 2), we found IOR at position S2 in the Respond condition; this IOR effect may reflect inhibition of sensory processing of targets following exogenous cues. However, IOR in the Ignore condition was found in a different position than that observed in the Respond condition. Namely, IOR was seen in the position of the first exogenous cue (S1/M1). The next experiment addresses one possible explanation for this discrepancy.

## EXPERIMENT 3

The findings so far can be summarized as follows: IOR appears either at position S2 or at position S1/M1, depending on whether subjects have to respond to the exogenous cues prior to localizing the target stimulus. When this initial series of responses to the cues is not required, IOR is only observed at S1/M1. Note that this procedure of ignoring exogenous cues and then responding to subsequent targets represents what is more or less the standard procedure in studies investigating IOR.

In the Ignore condition, the occurrence of IOR at S1/M1, but not at S2, is troubling since it suggests that IOR is stronger for an *earlier* cued location following multiple exogenous cues. This finding is at odds with other results[19,20] that have shown IOR to be greatest for the most recently cued position, being smallest or even eliminated for the location of the first cue. On the other hand, our observation of IOR at position S2 in the Respond condition is compatible with these previous findings, since *only* the most recently cued position showed IOR. Why should the patterns of IOR in the Respond and Ignore conditions be so different?

One possibility is that, somehow, subjects viewed the exogenous cues differently in the Respond and Ignore conditions, perhaps learning to suppress or alter their automatic response to the cues in the Ignore condition. There is evidence that people are capable of adjusting their attentional control settings[47] such that orienting responses to exogenous cues vary, depending on the attentional requirements of the task[5,22,48]. Similar arguments have been used to explain reduced exogenous cueing in a spatial cueing paradigm[49,50,51].

Certain factors could have influenced the development of such strategies in the Ignore condition. To begin with, the cues were spatially uninformative as to the location of the target. Furthermore, whereas in the Respond condition subjects had an explicit requirement that they recall where and in what sequence the cues occurred, there was no such requirement in the Ignore condition. Thus the cues were entirely irrelevant (except, perhaps, as time markers). Under these circumstances, subjects in the Ignore condition may have been strongly motivated to suppress their normal orienting responses to the exogenous cues. This would account for the disappearance of IOR at position S2. The remaining IOR at position S1 might be a residual effect of the initial cue at S1/M1 (important only perhaps for signaling the sequence to ignore), with orienting responses to the subsequent cue at S2 being suppressed.

Thus, one obvious possibility is that our findings in the Ignore condition, which contradict both our result from the Respond condition as well as those from comparable studies in the literature, was due to an alteration of the normal reflexive response to the exogenous spatial cues. To rule out this explanation, we carried out an additional experiment. In Experiment 3, we replicated the saccade task of Experiment 1. Subjects made saccadic localization responses to targets either (a) after replicating the cue sequence with a series of eye movements or (b) after passively viewing, but not responding to the cues. However, the testing conditions were altered in one important way. In Experiment 3, the Respond and Ignore trials were randomly mixed within a single session of trials. The third and final cue, i.e., the brightening of the fixation spot, gave the critical instruction informing subjects in what condition they were. When the fixation spot flashed from gray to green, subjects were instructed to “go”, making saccades to the cued locations in the correct order (Respond condition). When the fixation spot flashed from gray to red, they were instructed to “stay”, remaining focused on the fixation spot while waiting for the appearance of the target (Ignore condition). This type of color or shape fixation cue is frequently used in physiology experiments of various eye movement areas[52,53].

Since subjects did not know prior to the appearance of the third and final cue whether they would have to generate a series of responses to the cued locations, a reasonable assumption was that they could not ignore the cues. Thus, subjects could not alter their attentional control settings for an entire block of trials, but had to process the exogenous cues in exactly the same manner on both Respond and Ignore trials. We predicted that if a modulation of exogenous attention accounted for our peculiar results from the Ignore conditions of Experiments 1 and 2, then these results would not be observed under mixed trial presentation. In fact, the pattern of IOR in the Ignore condition should look more similar to that observed in the Respond condition, with IOR at the most recently cued position (S2). However, if IOR continues to be observed at S1/M1 in the Ignore condition, then it cannot be due to an alteration in how subjects are processing the cues.

## Methods

### *Participants*

Twenty-two students from the Rice University subject pool were tested in a single testing session. Subjects received partial course credit for participating. All had normal or corrected vision, and informed written consent (from both UT-Houston and Rice) was obtained from each participant. None of the subjects in Experiment 3 participated in the previous experiments.

### *Stimuli, Apparatus, and Procedure*

All experimental procedures were identical to those used in Experiment 1 with the exception of those described here. Rather than have two separate blocks of 128 trials for each of the Ignore and Respond conditions, a single block of 256 trials was given. Trials from the Respond and Ignore conditions were randomly intermixed. As before, the first two exogenous cues were flashed for 40 msec each at locations on the circular display separated by 90º. For the third and final cue, the fixation spot changed from gray to either green (signaling the Respond condition) or red (signaling the Ignore condition) for 40 msec, rather than white (as had been the case previously). Subjects were instructed that when the cue was red, they were to remain fixated during the ISI and make a saccade to the target as soon as it appeared. Following a green fixation cue, subjects were to make a series of eye movements to the cued positions in the correct sequence, and then remain at fixation until the target appeared. In all other respects, the procedures and criteria used were the same as those used in Experiment 1.

## Results

Each subject's median RT was calculated for targets appearing in each of the 8 possible positions, for both the Ignore and Respond conditions. Fig. 6 summarizes the mean of median reaction times for all subjects. A preliminary ANOVA with Condition (Respond/Ignore) and Target Position (1-8) as factors showed a significant effect of Condition, $F_{1,\,21} = 19.16$, $p < 0.0003$ (RTs of 368 and 404 for the Respond and Ignore conditions, respectively). Target Position showed a marginal effect ($F_{7,\,147} = 1.86$, $p < 0.08$). The Condition × Target Position interaction was not significant ($F_{7,\,147} = 1.11$, $p > 0.35$).

**Respond Condition – Planned Contrasts:** Planned contrasts were conducted using the Mean Square Error terms from separate ANOVAs of the Respond and Ignore conditions. In the Respond condition (MSE = 274.731), as before, there was significant inhibition at position S2 (8 msec, $F_{1,\,147} = 4.10$, $p < 0.05$; indicated in red in Fig. 6). In addition, there was no inhibition at position S1/M1 (3 msec, $F_{1,\,147} = 0.41$, $p > 0.52$), nor at either of the two motoric locations M2 (–4 msec, $F_{1,\,147} = 1.21$, $p > 0.27$) or M3 (1 msec, $F < 1$, $p > 0.78$). **Ignore Condition – Planned Contrasts:** For the Ignore condition (MSE = 394.249), responses to targets appearing at position S1/M1 were significantly inhibited (10 msec, $F_{1,\,147}$ =

4.38, $p < 0.04$; indicated in red in Fig. 6). There was no inhibition at position S2 (6 msec, $F_{1, 147} = 1.19$, $p > 0.27$). Finally, there was no hint of IOR at either position M2 or M3 (3 and 3 msec respectively, both Fs $< 1$, $p > 0.63$). As was the case in the previous two experiments, error rates were low and there were no significant effects (see Table 1).

## Discussion, Experiment 3

The results of Experiment 3 were essentially the same as that observed in Experiments 1 and 2. Different locations showed IOR, depending on whether subjects responded to or ignored the initial exogenous cues.

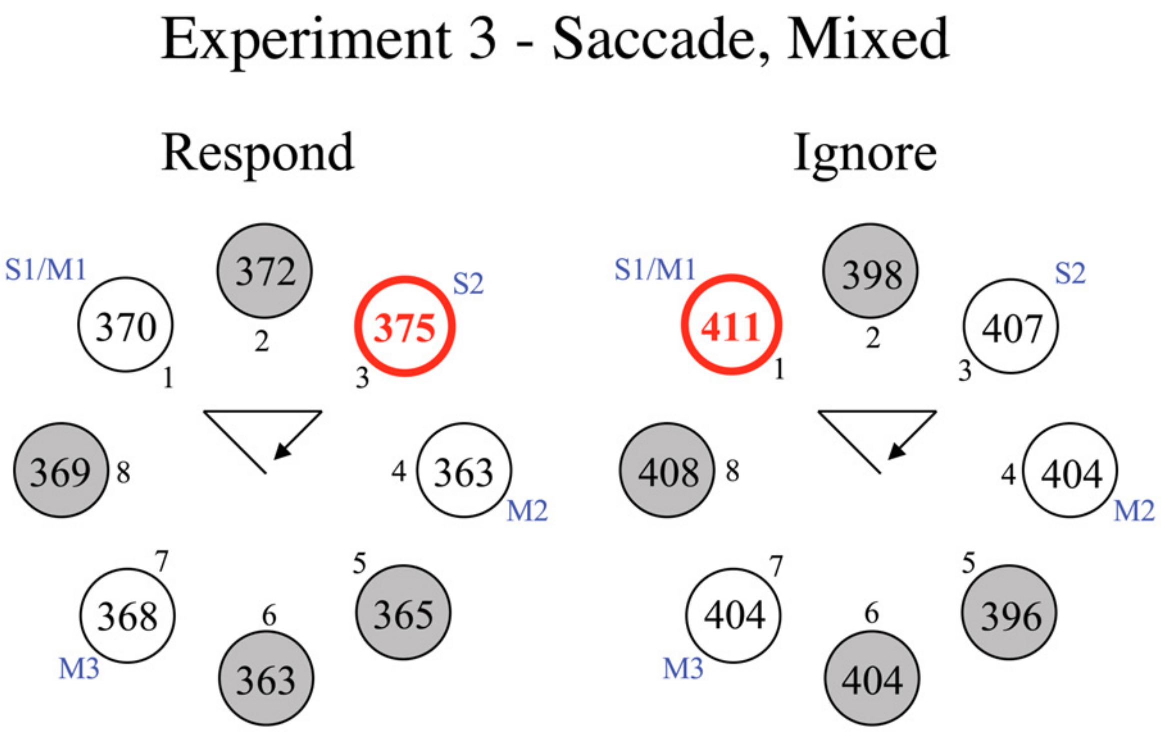

**FIGURE 6.** Mean of median saccadic RT in the Respond and Ignore conditions of Experiment 3. Again, formatting is the same as in Fig. 4.

Even mixing Respond and Ignore trials within a single block did not alter this pattern. Thus, it would appear that the particular IOR pattern we have observed in the Ignore condition can not be due to a strategy of blocking out or suppressing the normal responses to these exogenous cues via some type of modulation of attention or attentional control settings[22,47,50,51].

## EXPERIMENT 4

To better evaluate whether SOA played a role in explaining the Respond/Ignore differences, we conducted one additional experiment to address another possible explanation for our results so far. Specifically, the Respond and Ignore conditions resulted in different amounts of time elapsing between the initial cues and the subsequent target. For the Ignore condition, this SOA was fixed (1000 msec), but for the Respond condition, the effective SOA varied, depending on how long it took a subject to respond to the cues. Since it is known that SOA can affect IOR, it is possible that the different patterns of IOR observed in our Respond and Ignore conditions might have been affected by the actual amount of time that elapsed between the cues and the target. Thus, the net SOA between cues and targets in the Respond and Ignore conditions might potentially explain, for example, why no IOR was obtained in S1/M1 for the Respond condition, if any inhibition occurring at that position decayed during the added time delay.

A final experiment was conducted to match the SOA intervals between the Respond and Ignore conditions. Experiment 4 only presented the Ignore condition, but at two different SOAs. Two blocks of trials, called the Ignore Short and Ignore Long, were given. The Ignore Short trials were the same as in previous experiments, in that the target appeared 1000 msec after cue 3. The Ignore Long trial block replicated the timing sequence of the Respond condition, but did not require subjects to make saccades to the cued positions before viewing and responding to the target. In this block, the target appeared a total of 2670 msec after the onset of cue 3. This value is the average SOA between cue 3 and the target onset across all Respond conditions of Experiments 1, 2, and 3. The two Ignore conditions in this experiment thus matched the SOAs of the previous Respond and Ignore conditions in Experiments 1, 2, and 3. Any differences in IOR found to sensory or motor positions now reliably could be attributed to task, not SOA.

We predicted that, in the short SOA block of the Ignore condition, significant IOR would still occur only at S1/M1, replicating the pattern seen in Experiments 1, 2, and 3. Significant IOR for the long SOA block could either also occur only at S1/M1 or at no location if the elapsed SOA was long enough to eliminate IOR effects. However, we did not expect significant IOR to appear at S2 (similar to what was seen in the Respond condition of Experiments 1, 2, and 3) simply because SOAs were now equated. Not finding IOR at S2 in the Ignore Long condition would suggest that our previous observation of IOR in this position was due to task differences (specifically, due to the execution of the eye movement sequence) and not the difference in SOA.

### Methods

#### *Participants*

Each of the 18 students from the University of Texas-Houston or Rice University was tested in a single session consisting of two different experimental conditions. Subjects received monetary compensation for participating. All had normal or corrected vision, and informed written consent was obtained from each participant. None of the subjects in Experiment 4 participated in the previous experiments.

#### *Stimuli, Apparatus, and Procedure*

All experimental procedures were the same as Experiment 1 with the exception of those described here. Two counterbalanced blocks of the Ignore condition were presented. In one Ignore block, the target appeared 1000 msec after cue 3, just as had occurred in Ignore conditions of previous experiments. This block was called the Ignore Short block. In a second block of Ignore trials, called the Ignore Long block, the target appeared 2670 msec after cue 3.

## Results

Each subject's median RT was calculated for targets appearing in the 8 locations for both the Ignore Short and Ignore Long conditions. Fig. 7 shows a summary of the mean of all subjects' median reaction times. A preliminary ANOVA with Condition (Ignore Short/Ignore Long) and Target Position (1-8) as factors showed no significant effect of Condition, $F_{1,\,17} = 2.38$, $p > 0.14$ (RTs of 340 and 348 msec for the Ignore Short and Ignore Long conditions, respectively). Target position was not significant ($F_{7,\,119} = 1.73$, $p > 0.11$). The Condition × Target Position interaction was marginally significant ($F_{1,\,119} = 2.00$, $p < 0.06$).

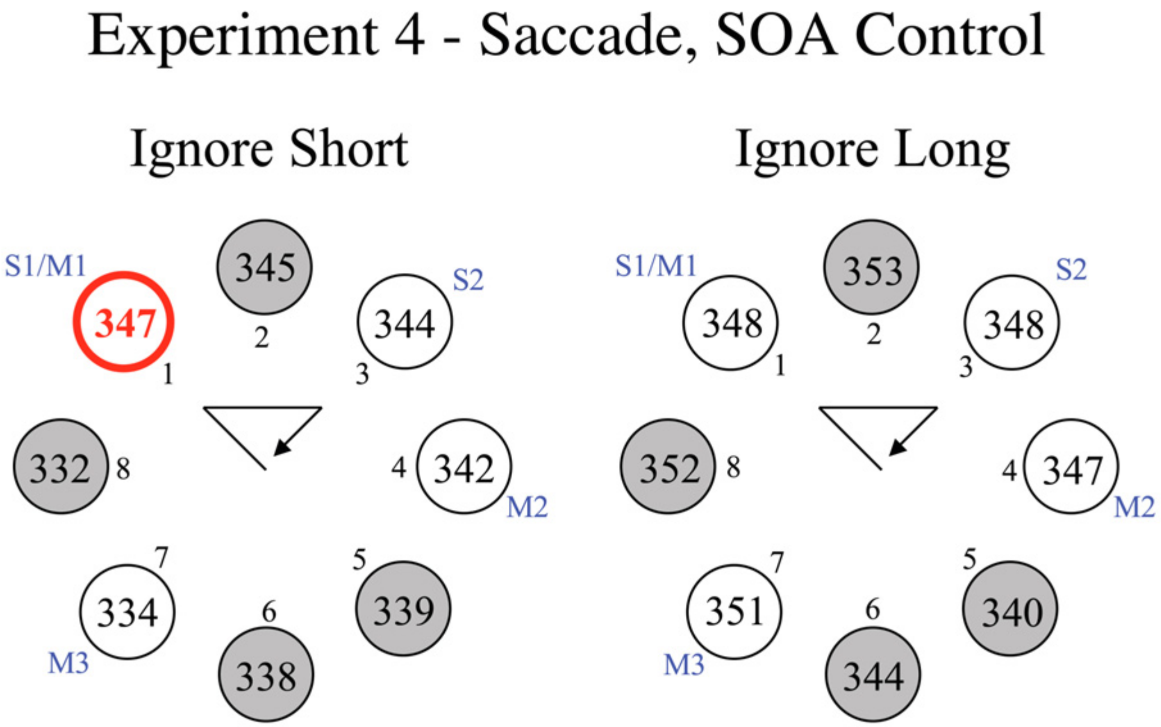

**FIGURE 7.** Mean of median saccadic RT in the Ignore Short and Ignore Long conditions of Experiment 4. Formatting is the same as in Fig. 4.

**Ignore Short Condition – Planned Contrasts:** Planned contrasts were conducted using the Mean Square Error terms from separate ANOVAs of the Ignore Short (MSE = 245.972) and the Ignore Long (MSE = 218.861) conditions. In the Ignore Short condition, as in previous Ignore conditions, there was significant inhibition at S1/M1 (8 msec, $F_{1,\,119} = 3.96$, $p < 0.05$; indicated in red in Fig. 7). IOR was not significant at S2 (5 msec, $F_{1,\,119} = 1.53$, $p > 0.22$). Neither M2 nor M3 had significant IOR (4 and –4 msec, $F_{1,\,119} < 1$ and $F_{1,\,119} = 1.04$, $p > 0.31$, respectively). **Ignore Long Condition – Planned Contrasts:** There was no evidence of IOR at any of the four positions (0, 0, –1, and 3 msec for S1/M1, S2, M2, and M3, respectively. All Fs < 1). Analysis of error data revealed that neither the main effects nor their interaction were significant. Planned contrasts on these also revealed no significant effects (see Table 1).

## Discussion, Experiment 4

As we had found in Experiments 1, 2, and 3, the Ignore Short condition (1000-msec ISI) showed a significant inhibition at S1/M1. However, no inhibition was found at any position in the Ignore Long condition, which had an equivalent SOA to the Respond condition of Experiments 1, 2, and 3. Specifically, no IOR occurred at S1/M1 in the Ignore Long condition, indicating this longer SOA was sufficient to eliminate the IOR effects found at S1/M1 in the Ignore Short condition. As we predicted, IOR was not found at S2 in the Ignore Long condition, suggesting that our previous observation of IOR in this position in Respond conditions was not due to the difference in SOA. These findings confirm that the differences in inhibition between the Respond and Ignore conditions of previous experiments are due to task differences.

## GENERAL DISCUSSION

Our main objective in this series of studies was to determine whether evidence for sensory or motor factors in IOR could be obtained within the same task and testing session. Previous evidence suggesting that sensory/attentional and motor processes each played a role in IOR was based on manipulations where the effects were revealed in different testing sessions or blocks of trials[13,15,21]. We had hoped that using a variation of a double-saccade task, we might be able to dissociate these two components of IOR within the same task and session. We summarize here the major empirical findings from these three experiments: (1) inhibition was observed for targets appearing at S2 (i.e., the location of the second peripheral cue), but only when subjects had to overtly respond to the preceding exogenous cues; (2) inhibition was observed at position S1/M1 (i.e., the position of the first peripheral cue), but only when subjects passively viewed and did not respond to the cues; and, (3) this pattern was the same for both saccadic and manual localization responses (see summary in Table 2). Below we consider the implications of and possible explanations for these data.

## Sensory IOR

When subjects had to repeat the cue sequence by making eye movements (Experiments 1 and 3) or by pointing (Experiment 2) to the cued locations prior to target onset, IOR was observed at position S2. As discussed earlier, IOR in this location is consistent with a sensory-based explanation of IOR, since no motor response from fixation towards position S2 was ever required. In the Respond condition, subjects had to orient to the cued positions sequentially prior to the appearance of the target. Observation of IOR under these circumstances is consistent with recent suggestions that IOR functions to enhance visual search[16,17,22,54] by preventing reorienting to previously inspected locations. However, it appears, based on the present findings, that it is not the repetition of prior orienting movements per se that is inhibited, but rather the detection of stimuli occurring at inspected locations. This is consistent with previous behavioral findings showing a strong sensory component[21] as well as recent neurophysiological recordings in the SC that report a reduced sensory response in neurons correlated with IOR[38], combined with a lack of any presaccadic effects in these neurons.

IOR was also observed at position S1/M1 in the Ignore condition. Although it may be parsimonious to argue that this IOR could have also been due to sensory factors, it could logically be due in whole or in part to motor factors. We address the evidence for and against motoric IOR in the next section.

## Motoric IOR

There was no unambiguous evidence for motoric IOR in the present experiments. Such evidence would have come from observing IOR in positions M2 or M3, which was not seen in any experiment. Instead, we consistently observed IOR at position S1/M1, but only in the Ignore condition. As we described earlier, IOR at this particular location could be due to sensory or motor factors.

However, the true nature of sensorimotor influences on IOR may be more complex than we have characterized them here. Rather than a clear distinction between processes that are purely sensory and those that are purely motoric, there may be some processes affecting IOR that do not fall clearly into one category or another. For example, although the majority of saccade-related neurons in the SC are not affected by visual stimulation, there are classes of saccade-related neurons in the SC that have a reduced or an absent response when saccades are made in the absence of a visual target[29,55]. Such partially or completely visually dependent saccade-related neurons are not purely motor, since they will not discharge (or else show a reduced discharge) before an eye movement when there is no visual target stimulus.

**TABLE 2**
**Comparison of median, mean, and geometric mean RTs for each condition of each experiment. Note that in all cases, where there are significant differences, the slowest RTs relative to the neutral conditions (indicated in boldface) are in positions S1/M1 for the Ignore condition and Ignore Short condition, and position S2 for the Respond condition.**

| | | Neutral | S1/M1 | S2 | M2 | M3 |
|---|---|---|---|---|---|---|
| **Experiment 1** | | | | | | |
| Respond | Median | 387 | 393 | **400** | 392 | 384 |
| | Mean | 397 | 400 | **407** | 402 | 391 |
| | Geo Mean | 392 | 395 | **401** | 396 | 388 |
| Ignore | Median | 358 | **370** | 364 | 357 | 355 |
| | Mean | 365 | **378** | 374 | 373 | 365 |
| | Geo Mean | 361 | **373** | 367 | 368 | 360 |
| **Experiment 2** | | | | | | |
| Respond | Median | 404 | 405 | **415** | 410 | 406 |
| | Mean | 416 | 419 | **426** | 425 | 416 |
| | Geo Mean | 411 | 414 | **421** | 419 | 411 |
| Ignore | Median | 392 | **401** | 389 | 391 | 391 |
| | Mean | 401 | **409** | 400 | 404 | 403 |
| | Geo Mean | 397 | **404** | 396 | 399 | 397 |
| **Experiment 3** | | | | | | |
| Respond | Median | 367 | 370 | **375** | 363 | 368 |
| | Mean | 376 | 381 | **391** | 373 | 376 |
| | Geo Mean | 371 | 376 | **385** | 370 | 372 |
| Ignore | Median | 401 | **411** | 407 | 404 | 404 |
| | Mean | 410 | **417** | 415 | 414 | 414 |
| | Geo Mean | 404 | **411** | 410 | 407 | 407 |
| **Experiment 4** | | | | | | |
| Ignore Long | Median | 347 | 348 | 348 | 347 | 351 |
| | Mean | 352 | 353 | 351 | 348 | 355 |
| | Geo Mean | 353 | 352 | 353 | 350 | 356 |
| Ignore Short | Median | 338 | **347** | 344 | 342 | 334 |
| | Mean | 346 | **357** | 349 | 352 | 342 |
| | Geo Mean | 347 | **357** | 356 | 353 | 347 |

If these types of saccade-related SC neurons mediate motoric IOR, then maintenance of IOR might depend critically on the continued presence of visual stimuli at the cued locations. Just such a population of cells possibly could explain why IOR observed in search paradigms seems to depend on the maintenance of the search array[16,54]. Cells with these characteristics could allow for the possibility of a visually dependent motor-based IOR.

## Motoric IOR at S2?

While we have argued that IOR at position S2 must be sensory (see above), it could be argued that eye movements are automatically programmed towards S2 as a result of the exogenous cue, even though no saccades from fixation towards that position are made. If this was the case, then perhaps the IOR seen at S2 reflects not deficient sensory processing but, instead, an inhibition of previously generated, but cancelled motor responses towards that position. Based on the present data, there are two reasons why we think such a scenario is unlikely.

First, in the Respond condition, the subject executes three eye movements (or pointing responses) before the target appears. We predicted that the planning and execution of these three movements would lead to inhibition of subsequent responses towards M1, M2, and M3 (see the Statistical Analysis section in the Methods section of Experiment 1). However, in the Respond condition, none of these three positions demonstrated IOR. We feel it unlikely that planned-but-cancelled responses towards S2 would result in inhibited responses, while planned-and-executed responses would result in no inhibition of responses at M1, M2, or M3. Further, any automatic programming of a saccade towards S2 would have been followed by the subsequent preparation (and execution) of these three eye movements (M1, M2, and M3). Thus, any motoric IOR at S2 would have been susceptible to additional decay (compared to M1, M2, and especially, M3). Hence, we feel it is unlikely that IOR at S2 is due to saccadic programming, since saccade programs generated subsequent to the cues did not lead to IOR.

A second reason why we believe that motoric factors are not responsible for IOR at S2 is that in the Ignore Condition, there is no inhibition at this position. If the cue at S2 automatically generates a movement program, this should be true in both the Respond and the Ignore conditions. In fact, in the Ignore condition, any automatically programmed saccade to S2 would not be followed by subsequent programming of these three other saccades and the additional delays that accompany those responses. Thus, due to issues of recency, motoric IOR at S2 should be greater in the Ignore condition than in the Respond condition. Yet in the Ignore condition, there is no IOR at S2. Based on this, any IOR occurring at S2 must be due to sensory factors and is unlikely to be caused by programming of saccades towards S2.

## Spatiotopic IOR at S2 in the Respond Condition?

An alternative account of IOR at location S2 is a spatiotopic or environment-based IOR[56]. The Respond condition is similar to recent studies using visual search paradigms showing that responses are slower to targets presented in the region of the previous fixation[57]. This spatiotopic account differs from the retinotopic sensory cue-driven account that forms the basis of the present set of experiments. As briefly discussed in the Introduction, the SC, as well as other cortical areas considered important for eye movements and spatial representation, contains an orderly retinotopic representation, i.e., activity depends on the vector from the current eye position to the cue or movement end point location[29,30,31,32]. After the execution of an initial saccade, the loci of activity within these areas are remapped to bring them back into registration with the new retinal positions of the stimuli[58,59,60]. It is this remapping activity that is the neural correlate of a spatiotopic reference frame that is remapped onto a retinotopic reference frame. For some units in these areas, remapping occurs before the onset of the saccade. This activity typically has been demonstrated to occur preceding the next movement, not throughout the entire sequence of movements. Specifically, neurophysiological studies have shown that when a sequence of responses are executed, movement-related neurons in both the SC and lateral intraparietal cortex (LIP) normally show activity only for the next planned movement, not for the entire sequence[29,60,61,62]. Thus, if a series of saccades from A to B to C is planned, neural activity is first observed reflecting the movement toward location A. Neural activity reflecting movement toward location B is seen only after the initial movement to A has been executed.

The brain regions in which remapping have been demonstrated (e.g., LIP, FEF, SC) are all strongly involved in the control of eye movements. Recent work suggests, however, that there is also remapping in early, more strictly sensory, extrastriate visual areas such as V2, V3, and V3a[63]. Although some studies examining IOR have distinguished between a retinotopic and spatiotopic reference frame, the majority of work has been performed within a retinotopic reference frame. Hence, it remains to be clarified to what extent IOR characteristics (e.g., sensory/motor effects, time course, task demands) are similar in both frames of reference.

## IOR at the Most Recent Location?

An unexpected and troubling aspect of the present data is the pattern of IOR observed in the Ignore condition where we found IOR at only one location following multiple cues. In this case, IOR was seen not at the most recently cued peripheral location, but instead at the location cued the earliest. This is obviously in conflict with all studies of IOR including recent studies using multiple exogenous cues showing maximum IOR at the most recently cued location[19,20]. As we noted previously, this procedure of ignoring exogenous cues and then responding to subsequent targets is commonly used in studies investigating IOR. Therefore, we will address this aspect of our data in some detail.

One factor that may have contributed to our observation of IOR at the first cued position is the particular type of cue sequence we used. In other studies that have investigated IOR using multiple cues[19,20], subjects were presented with a variable number of cues that also appeared in randomly chosen locations or orders. In contrast, the procedure we used was very different. We chose to reduce the number of possible sequences instead of randomly selecting from the set of all those possible (56 cue sequences, 448 cue-target sequences). The 16 cue sequences (128 cue-target sequences) we actually used were constrained by our need to reduce the number of trials required for a fully balanced design, and by our desire to use sequences which would potentially allow IOR to be observed in specific positions relative to those cues (i.e., see Fig. 3). Thus, we only used two peripheral cues, and these were always separated in the display by 90º. As soon as subjects viewed the initial exogenous cue the position of the next one was highly predictable (i.e., it was either 90º clockwise or counterclockwise in the display).

We have personally observed these types of cue sequences many thousands of times in the course of setting up and conducting these experiments. Based on our own observations and reports from participants, the cues, under the viewing conditions described, do not appear as individual independent events or stimuli, but rather as part of a unified sequence or group. The use of multiple exogenous cues of this type is virtually unreported in the literature, and hence they may operate in a manner very different from those typically employed in studies of spatial orienting, and have very different consequences. It may be, for example, that when shown highly constrained patterned cues such as those employed here, subjects reorient to the location specifying the beginning of the sequence (see also, in support, above discussion on the neurophysiology of eye movement sequences). This would account for the fact that IOR subsequently was observed only at S1/M1 in the Ignore condition.

It is important to note that this proposed explanation does not conflict with the findings in the Respond condition. In particular, the fact that subjects in the Respond condition did not show IOR at the first cued position should not be taken to imply that the cues in this condition were not perceived as a unified sequence, whereas they were perceived as such in the Ignore condition. Most likely they were perceived in a similar manner in both conditions. And, indeed in Experiment 3, which involved mixed presentation of Respond and Ignore trials, it would have been quite impossible for the cues to be perceived differently in these conditions. Instead, as discussed above (in the section on spatiotopic IOR), the requirement that subjects overtly respond to the cues in the Respond condition had the effect of lengthening the time interval from initial perception of the cue sequence, per se. Experiment 4 demonstrates that this additional time interval between the cue sequence and target response is sufficient to eliminate the inhibition at S1/M1. Hence, in the Respond condition, even though subjects reoriented to the first cued position, they then executed the three movements. Experiment 4 also demonstrates that it is not the additional time interval that induces inhibition at S2 in the Respond condition, but rather the execution of three movements. Under these conditions, the situation in our Respond condition is more akin to a visual search paradigm, where it has been shown that IOR accrues to recently searched locations[16].

While this explanation is admittedly an ad hoc explanation, it is worth noting that numerous studies have demonstrated that IOR can be eliminated or reduced by changing various task demands: for example, (1) identification vs. detection tasks[5,64]; (2) direct vs. indirect stimulus-response mapping[6]; and, (3) task difficulty[22,65]. Our present findings, regarding the possible effects of the perception of a sequence of events on IOR as well as the effects of responding to the cues on IOR effects at the most recently searched location (S2), may suggest additional important boundary conditions that will be critical to understanding the mechanisms underlying IOR.

## Source of IOR: Cues and/or Movements

As we indicated in our introduction to Experiment 1, previous work demonstrated that IOR can be generated after presentation of a peripheral cue or after a peripheral cue with response. Only two previous studies directly compared these two types of cueing procedures and both showed an enhanced IOR when subjects responded to the cues[15,21]. We now have also made this comparison in a paradigm employing multiple cues. Our initial rationale for including this manipulation was to enhance our chances for dissociating robust motor and/or sensory influences and effects on IOR. Unexpectedly, we report consistent significant differences between a cue only (Ignore) and cue with response (Respond) condition in three experiments. Given that previous work has not reported differences in the presence of IOR between these conditions, we believe our multiple cue paradigm is responsible for eliciting such differences. It is possible that physiologically separate IOR processes are generated due to cues or movements, or some combination thereof. In addition, there may be physiologically separate IOR processes depending on whether the cue or movement are voluntary (endogenous) or reflexive (exogenous). Similar distinctions (both behavioral and physiological) have now been established between the attentional effects that arise from a peripheral noninformative cue and a central symbolic cue[50,66,67,68,69,70]. These distinctions took many years to establish. Further, we have suggested that the unusual findings of IOR at the first (not last) cued location in the Ignore condition suggest that mental schema (i.e., the perception of a sequence or grouping of the multiple cues) can fundamentally affect IOR. Future studies will be needed to test these ideas. It will take more experiments to sort out the possible sources for these findings.

## Eye Movements vs. Pointing

One striking aspect of our data is that the results were essentially the same for both saccadic and manual localization responses. Unlike one recent study[71], we found no difference between IOR effects for saccadic and manual responses. We believe it likely that this is due to the fact that in both of the present experiments, we employed stimulus-directed responses. Elsewhere, we have obtained a similar pattern of synchrony when saccadic responses are compared to pointing responses[6], but not when more abstract manual responses are considered (e.g., key press)[3], such as the response employed by Abrams and Pratt. We proposed[6] that these more indirect responses critically involve other areas in prefrontal cortex.

As we indicated above, there has been very little use of manual reaching or pointing responses in the study of IOR[72,73]. Thus, our observation of virtually identical results from saccadic and manual versions of the same target localization task would be a significant empirical contribution to the IOR

literature if proven reliable. The one caveat on this point is that we did not monitor eye movements when subjects in the present experiments were performing the manual task. Our intuition based on observation of some subjects (and performance of this task ourselves) is that subjects do not tend to make eye movements during the manual task, because doing so hinders efficient performance. We previously showed that manual IOR effects obtained with pointing tasks similar to that used in the present study are identical, whether subjects move their eyes or not[6]. Hence, we do not think that eye movements during the pointing task would necessarily have affected the results, even if they did occur. The similarity of the pattern of IOR effects for saccadic and manual stimulus-directed responses would be entirely consistent with the proposal that the SC is implicated in IOR, given recent evidence that some collicular neurons are activated during goal-directed arm movements[43,44,45,46].

## CONCLUSIONS

Much behavioral work has focused on distinguishing sensory- and motor-based components of IOR[15], as well as other higher-order cognitive processes. A similar question has frequently been raised in physiology. Two brain areas thought to act as interfaces between sensory and motor processing are the posterior parietal cortex and the SC; and the debate over the relative importance of sensory and motor influences on activity in these brain areas is long standing and well documented[29,74,75]. We employed a variation of a double-saccade task, a procedure utilized by researchers to dissociate sensory and motor influences on activity in SC neurons in order to dissociate sensory/attentional and motor factors in IOR. With this procedure, we obtained three main findings. First, IOR occurred when subjects overtly responded to a series of exogenous cues. Although the inhibited location was previously stimulated by a peripheral cue and, hence, could be interpreted as a sensory-driven IOR effect, we believe the effect in our experimental design was driven by the required sequence of responses that followed the cues and the inhibitory effect was greatest for the last attended and searched spatial location. Hence, subjects were inhibited from returning to the most recently searched location. Second, IOR occurred when subjects passively viewed the cues, but, in this case, it was seen at the position that was cued first. This IOR could have been due to either motor or sensory processes. The observation of IOR at the first as opposed to the most recently cued position is surprising, and the conditions under which earlier rather than later cued positions exhibit IOR merits further investigation. We believe that the perception of multiple cues in our study as a unified group or sequence caused subjects to automatically reorient to the location at the beginning of the sequence and, hence, influenced the observation of IOR. Finally, the same pattern of IOR was observed for both oculomotor and manual responses when these responses involved direct stimulus-response mappings or goal-directed responses. The similarity of data from saccadic and pointing versions of the same task should serve to emphasize the importance of controlling response factors in studies of IOR, a point that we have made elsewhere[6]. Taken as a whole, the present results suggest that there may be multiple sources for the effects of IOR and that these effects are sensitive to our perceptual organization of the world.

## ACKNOWLEDGMENTS

The authors would like to thank Nnenna Agim and Julia Homolova for help in data collection, Shelly Babin for comments on an earlier version of this manuscript, Cate Cathey for assistance with manuscript preparation, and Lorenzo Morales for help in creating and optimizing figures. This work was supported in part by grants from the James S. McDonnell Foundation, NARSAD Essel Investigator, and NIH (R01 MH63340 and R01 MH65492) grants to A.B. Sereno, by a NARSAD grant to K.A. Briand, and an NIH Neuroscience Training Grant (5 T32 NS07467) to C.B. Jeter.

## BIOSKETCHES

**Anne B. Sereno, PhD,** is an Associate Professor in Neurobiology and Anatomy at the University of Texas-Houston Medical School and an Adjunct Associate Professor in Psychology at Rice University. E-mail: anne.b.sereno@uth.tmc.edu

**Cameron B. Jeter** is a graduate student in the Department of Neurobiology and Anatomy at the University of Texas-Houston Medical School.

**Vani Pariyadath** is a graduate student in the Department of Neurobiology and Anatomy at the University of Texas-Houston Medical School.

**Kevin A. Briand, PhD**, is a Research Specialist in the Department of Research and Accountability in the Houston Independent School District and is a postdoctoral fellow in the Department of Neurobiology and Anatomy at the University of Texas-Houston Medical School.
